# Functional Analysis of the *Phycomyces carRA* Gene Encoding the Enzymes Phytoene Synthase and Lycopene Cyclase

**DOI:** 10.1371/journal.pone.0023102

**Published:** 2011-08-09

**Authors:** Catalina Sanz, Antonio Velayos, María Isabel Álvarez, Ernesto P. Benito, Arturo P. Eslava

**Affiliations:** 1 Department of Microbiology and Genetics, University of Salamanca, Salamanca, Spain; 2 Centro Hispano-Luso de Investigaciones Agrarias, University of Salamanca, Salamanca, Spain; Oregon State University, United States of America

## Abstract

*Phycomyces carRA* gene encodes a protein with two domains. Domain R is characterized by red *carR* mutants that accumulate lycopene. Domain A is characterized by white *carA* mutants that do not accumulate significant amounts of carotenoids. The *carRA*-encoded protein was identified as the lycopene cyclase and phytoene synthase enzyme by sequence homology with other proteins. However, no direct data showing the function of this protein have been reported so far. Different *Mucor circinelloides* mutants altered at the phytoene synthase, the lycopene cyclase or both activities were transformed with the *Phycomyces carRA* gene. Fully transcribed *carRA* mRNA molecules were detected by Northern assays in the transformants and the correct processing of the *carRA* messenger was verified by RT-PCR. These results showed that *Phycomyces carRA* gene was correctly expressed in *Mucor*. Carotenoids analysis in these transformants showed the presence of ß-carotene, absent in the untransformed strains, providing functional evidence that the *Phycomyces carRA* gene complements the *M. circinelloides* mutations. Co-transformation of the *carRA* cDNA in *E. coli* with different combinations of the carotenoid structural genes from *Erwinia uredovora* was also performed. Newly formed carotenoids were accumulated showing that the *Phycomyces* CarRA protein does contain lycopene cyclase and phytoene synthase activities. The heterologous expression of the *carRA* gene and the functional complementation of the mentioned activities are not very efficient in *E. coli*. However, the simultaneous presence of both *carRA* and *carB* gene products from *Phycomyces* increases the efficiency of these enzymes, presumably due to an interaction mechanism.

## Introduction

**Figure 1:**
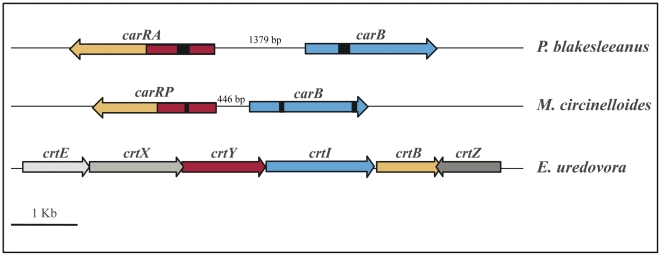
Schematic organization of carotenogenesis structural genes. The organization of the structural genes involved in the carotenoid biosynthesis pathway in *P. blakesleeanus*, *M. circinelloides* and *E. uredovora* is shown. The function of these genes is described in the main text.

**Figure 2:**
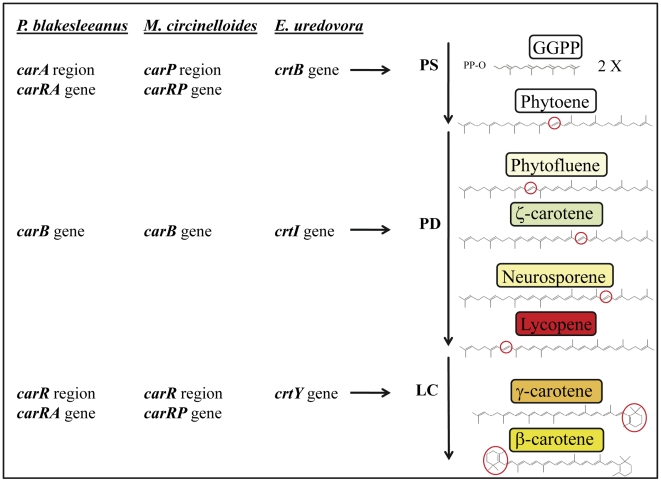
Carotenogenesis pathway. Enzymatic steps and structural genes involved in the biosynthesis of β-carotene from GGPP in *P. blakesleeanus*, *M. circinelloides* and *E. uredovora*. PS, phytoene synthase; PD, phytoene dehydrogenase; LC, lycopene cyclase.

Carotenoids are natural pigments widely distributed that contribute to the color of many plants and animals and play a major role in photoprotection. Carotenoid derivatives are of great importance in different processes such as vision, nutrition, cellular growth and development. Biosynthesis of carotenoids occurs in all photosynthetic organisms as well as in many nonphotosynthetic bacteria and fungi [1]–[3]. *Phycomyces blakesleeanus* accumulates ß-carotene, a C40 carotenoid which gives this fungus its typical yellow color [4]. The biosynthetic pathway of all C40 carotenoids starts with the condensation of two geranylgeranyl pyrophosphate (GGPP) molecules to form the colorless compound phytoene, a step catalyzed by the enzyme phytoene synthase. From this point, the pathway can take different directions depending on the organism [5]. In *Phycomyces*, phytoene is converted into lycopene by four consecutive dehydrogenation reactions carried out by the enzyme phytoene dehydrogenase. Finally two cyclizations at both ends of the lycopene molecule catalyzed by the enzyme lycopene cyclase give rise to ß-carotene, the end-product of the pathway (Figures 1 and 2). These enzymes are thought to be organized in an aggregate, formed by four and two units of phytoene dehydrogenase and lycopene cyclase, respectively, that works as an assembly line [6]–[10].

The study of carotenogenesis in *Phycomyces* is facilitated by the large number of mutants available, both in structural and regulatory carotenogenic genes, and the easiness in obtaining them. The use of classical genetics techniques such as complementation, including quantitative complementation [6], [10]–[12], and genetic sexual analysis [13]–[17], as well as the existence of a general model for carotene biosynthesis and its regulation [18], [19] that allows testing hypotheses, constitute important tools in this field. The main downside of this system comes from the failure to obtain stable transformants with exogenous DNA in *Phycomyces* [20]. This problem is compensated in part by using the related fungus *Mucor circinelloides*, which can be easily transformed and is more amenable to molecular techniques [21] and where *Phycomyces* genes can be expressed [22], [23]. Moreover, both fungi seem to have a very similar carotenogenic pathway (Figures 1 and 2) although with clear differences in its regulation [24]. In *Phycomyces* this pathway is feed-back regulated by the end-product, while in *Mucor* and *Blakeslea* such a regulation is absent or clearly different [4], [25].

In *Phycomyces* the biosynthesis of β-carotene is influenced by external agents such as culture conditions, chemicals, blue light and sexual interactions [25]. The activation of the β-carotene biosynthesis by light requires the products of genes *madA* and *madB*, which are components of a photoreceptor and transcription factor complex [26], [27] that is homologous to the White Collar complex in *Neurospora crassa* [28], [29]. Protein binding complexes are suggested to be involved in the down-regulation of photocarotenogenesis in *Phycomyces* [30]. Despite the differences in the pathway regulation, the similarities between the structural genes of both organisms make *Mucor* a system suitable to test the function of the *Phycomyces* genes involved in the synthesis of carotenoids [23].

Among the many carotenoid mutants isolated in *Phycomyces*, those from a particular class, obtained after a single mutagenic treatment, seemed to lack two functions controlled by a single gene, denoted as *carRA*, because they did not complement mutants belonging to two different complementation groups (*carR* and *carA*) [31]. *carR* mutants are red because they accumulate lycopene, and are supposed to be altered at the lycopene cyclase, while *carA* mutants are white, accumulate only traces of β-carotene, and were supposed to be altered in substrate transfer in and between the carotenogenic enzyme complexes [31], [32]. Similar mutants were also described in *M. circinelloides* and the gene was termed *carRP* [33]. Biochemical analysis of a particular *Mucor* strain later shown to be a *carRP* mutant suggested an alteration in the synthesis of phytoene [34]. Isolation and characterization of the *Mucor carRP* gene (AJ250827) confirmed this hypothesis, showing that both lycopene cyclase and phytoene synthase activities are encoded by this gene [35]. The same situation had previously been reported by Verdoes et al. [36] for gene *crtYB* (AJ133646) in the basidiomycetous yeast *X. dendrorhous*. Another previously isolated fungal gene, *al-2* (L27652) from the ascomycete *Neurospora crassa*, initially reported as the phytoene synthase gene [37], was then also proved to encode the lycopene cyclase activity [38], [39]. The enzymatic activities encoded by these genes (*crtYB*, *carRP*, and *al-2*) have been proven by complementation assays in *E. coli* containing plasmids carrying different carotenogenic genes from *Erwinia uredovora* [40].

The isolation of the *Phycomyces carRA* gene (AJ276965), the detection of changes in its sequence in several mutants, and the correlation with homologous sequences from the above mentioned fungi has led to identify it as the gene encoding not only the lycopene cyclase activity but also the phytoene synthase [41] (Figures 1 and 2). Unlike for its homologous genes listed above, however, no functional data have been reported so far. In this work we undertook detailed functional analyses of the *Phycomyces carRA* gene in the heterologous systems *M. circinelloides* and *E. coli* to confirm the molecular nature of the activities codified by this gene as was previously done for the other structural gene of this pathway in *Phycomyces*, the *carB* gene (X78434) [23], [42].

## Materials and Methods

### Plasmids, strains and growth conditions

The plasmids used in this work and their main properties are listed in Table 1. Vector NTI Suite software package (InforMax, North Bethesda, Md., USA) was used for managing and analysis of plasmids and other DNA sequences. *E. coli* strain DH5α was used for all cloning experiments and plasmid amplifications, and was grown at 37°C in Luria broth (LB) medium appropriately supplemented [43]. *P. blakesleeanus* wild type (NRRL1555) was grown at 22°C on minimal medium (SIV) [44]. *M. circinelloides* wild-type (CBS277.49) and mutant strains MS7 (*leuA1, carP4*), MS8 (*leuA1, carRP5*) and MS21 (*leuA1, carR9*) [33] were grown at 22°C on minimal (YNB) and complete (YPG) media, with the pH adjusted to 4.5 for normal or 3.0 for colonial growth, and supplemented with leucine (200 µg/mL) when needed [45].

**Table 1:** Plasmids used in this work.

PLASMID	RELEVANT CHARACTERISTICS^a^	REFERENCE
pUC19	*Amp*	[53]
pKS+	*Amp*	Stratagene
pAVB2	pUC19 : *crtY* gene	[35]
pAVB5	*crtE*, *crtI*, *crtY* genes (phytoene synthase mutant). *Cm*	[35]
pAVB12	*crtE*, *crtB*, *crtI* genes (lycopene cyclase mutant). *Cm*. Lycopene production	[35]
pAVB13	pUC19 : *crtB* gene	[35]
pAVB16	*crtE*, *crtI* genes (phytoene synthase/lycopen cyclase double mutant). *Cm*	[35]
pCS19	pUC19: *P. blakesleeanus carRA* cDNA	This work
6pCS16	*crtE* gene. *P. blakesleeanus carB* cDNA. *Cm*	This work
pCS5.1(3)	pKS+: *P. blakesleeanus carRA* and *M. circinelloides leuA* genes	This work

a *Erwinia uredovora* genes are: *crtE* (geranylgeranyl pyrophosphate synthase), *crtB* (phytoene synthase), *crtI* (phytoene dehydrogenase), and *crtY* (lycopene cyclase). *Cm*: chloramphenicol resistance. *Amp*: ampicillin resistance. *P. blakesleeanus carRA* and *carB* cDNAs in pCS19 and 6pCS16 plasmids are cloned in the multiple cloning site of vector pUC19.

### Preparation and transformation of *M. circinelloides* protoplasts

**Figure 3:**
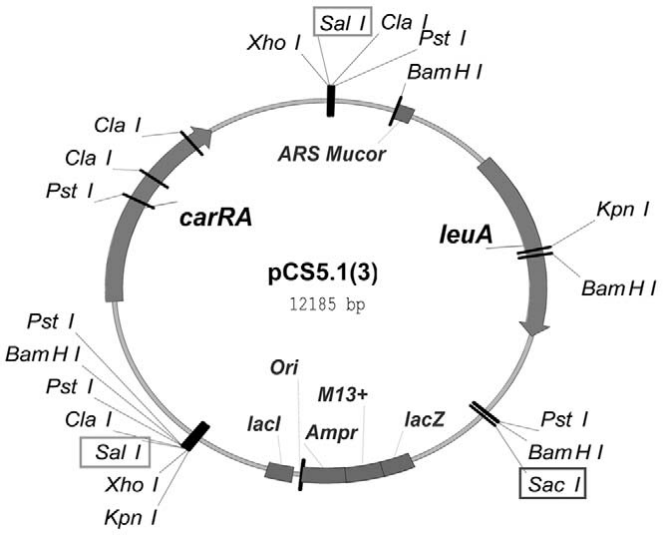
Restriction map of plasmid pCS5.1(3). This plasmid includes the *M. circinelloides leuA* and the *P. blakesleeanus carRA* genes. It was employed in the transformation of the *M. circinelloides* strains MS7, MS8 and MS21. Digestion with *Sac*I results in plasmid linearization (12-kb single band). Digestion with *Sal*I gives rise to two fragments of 7.3 kb (including the *leuA* gene) and 4.7 kb (including the *carRA* gene).

Protoplasts of leucine auxotrophic *M. circinelloides* strains MS7, MS8 and MS21 were prepared by treatment of washed germlings with 1.5 mg/ml Novozyme_234_ (Novo Industries, Denmark) and 4–12 U/ml of Streptozyme in the presence of 0.5 M sorbitol and 10 mM sodium phosphate buffer, pH 6.5 [22]. Transformation of protoplasts was polyethyleneglycol (PEG)-mediated [21]. Transformations were performed with plasmid pCS5.1(3) (Figure 3) containing the genomic copies of the *P. blakesleeanus carRA* and the *M. circinelloides leuA* genes, and prototrophic transformants were selected on minimal medium. Several transformants from each mutant strain were transferred individually to fresh minimal medium plates and incubated under continuous light for four days.

### Purification of nucleic acids

The isolation of genomic DNA from *P. blakesleeanus* and *M. circinelloides* was performed as previously described [10], [46]. Total RNA was isolated by the guanidine isothiocyanate method [47], except that the mycelium was ground with pestle and mortar while frozen. *Mucor* and *Phycomyces* strains were cultured in minimal medium plates supplemented as required and grown for 4 days at 22°C and under continuous broad-band blue light irradiation at a fluence of 40 J/m^2^ (4 W/m^2^ for 10 s).

### Standard molecular procedures

Commonly used protocols for plasmid DNA purification, cloning, transformation of *E. coli*, and electrophoresis and transfer of DNA onto nylon filters were followed [43]. DNA fragments for subcloning or labeling were recovered from agarose gels and purified using the Geneclean kit (BIO 101, Ohio, USA). Total RNA was electrophoresed in MOPS/formaldehyde buffer and transferred to a nylon membrane. Labeling, hybridization and immunological detections were carried out using the “non-radioactive labeling and immunological detection kit” and the “polymerase chain reaction (PCR) digoxigenin labeling mix” (Roche, Indianapolis, USA) following the supplier's recommendations. For Southern analyses, a *carRA* gene derived probe was labeled by PCR using oligonucleotides PSP8 (5′-CAAAGGAGAACACGGAAG-3′) and PSP10 (5′-TGCAAAGGCCTGGGTATG-3′). A *leuA* probe was generated by random primed labeling. For Northern analyses, a *carRA* probe was obtained by PCR using total cDNA and oligonucleotides PSP12 (5′-ATCTTACTCGAGGATGCTGACTTATATG- 3′) and PSP13 (5′-TTTTGAGCTCTTAAATGACAGTAAAGGC - 3′). PCR amplification conditions were performed as previously described [10]. After autoradiography, the films were analyzed with an Image Acquisition Console and the Whole Band Analyzer software (Bio Image; MilliGen/Biosearch, Ann Arbor, Mich., USA).

For RT-PCR analysis, synthesis of total cDNA and amplification reactions were carried out in a Thermal Cycler 480 (Applied Biosystems, N.J., USA). For each cDNA synthesis reaction, 1 µg of total RNA was mixed with 1 µl of oligonucleotide d(T)_16_ (50 µM), incubated at 70°C for 10 min and cooled on ice before adding 4 µl of 5× PCR buffer, 2 µl of DTT 0.1 M and 8 µl of 2.5 mM of each dNTP. The mix was incubated at 42°C for 2 min before addition of 1 µl of SUPERSCRIPT™ II RNAse H Reverse Transcriptase at 200 U/µl (LIFE TECHNOLOGIES, California, USA). Reactions were incubated at 42°C for 50 min, and then inactivated at 70°C for 15 min. For the amplification of the *P. blakesleeanus carRA* cDNA, oligonucleotides PSP8 (5′-CAAAGGAGAACACGGAAG-3′) and PSP14 (5′-AAGGCCTGGGCCAGCAG- 3′) were used. The sequence of PSP14 comprises 9 nucleotides of the first exon 3′ end followed by 8 nt of the second exon 5′ end, so the amplification with PSP8 and PSP14 gives rise to a 510 bp fragment of the cDNA obtained after the splicing of the *carRA* intron. In addition, to confirm the quality of the cDNA synthesis reactions, a control PCR was performed to amplify the *pyrG* gene cDNA using oligonucleotides pyrG3 (5′-ATGATGCTGAACACATACAAG-3′) and pyrG4 (5′-TTATGCTTTATGCATGCTTAC-3′). The amplifications were performed in 100 µl reactions containing 2 µl of the cDNA synthesis reaction, 0.15 µM of each primer, 2 mM MgCl_2_, 200 µM of each dNTP, 1× PCR buffer II and 2.5 U of AmpliTaq DNA polymerase (Applied Biosystems, California, USA) with the following PCR profile: 95°C for 2 min; 40 cycles of 95°C for 30 s, 55°C for 1 min and 72°C for 90 s; 72°C for 5 min.

### Carotenoid analyses

Determination of carotenoids accumulated in both *M. circinelloides* and *E. coli* was performed by HPLC analysis as previously described [35].

## Results

### The *Phycomyces carRA* gene complements mutations in *Mucor carRP*

In order to confirm that the *carRA* gene codes for an enzyme with lycopene cyclase and phytoene synthase activities, as the *M. circinelloides carRP* gene does, functional complementation in *M. circinelloides* was attempted. Plasmid pCS5.1(3) (Figure 3) carrying genomic DNA copies of the *P. blakesleeanus carRA* and the *M. circinelloides leuA* genes was employed to transform the *M. circinelloides* strains MS7 (*leuA1*, *carP4*), MS8 (*leuA1*, *carRP5*) and MS21 (*leuA1*, *carR9*). MS7 and MS8 strains are white because they do not accumulate any carotenoid; MS21 strain shows a pink phenotype due to accumulation of lycopene. Approximately 20 transformants per experiment were obtained for each of the three mutant strains. Several *leu+* transformants derived from each mutant strain were transferred individually to fresh minimal medium plates and incubated under continuous light conditions for four days. No clear differences among the transformants for each strain could be detected by visual inspection suggesting a similar degree of complementation. For further analysis we selected three transformants of each type. Visual inspection of mycelia color indicated that MS7 and MS8 transformants showed a light-creamy color, slightly darker than the clean white color characteristic of the untransformed MS7 and MS8 strains. The MS21-derived transformants showed a slightly orange color somewhat distinguishable from the typical pink color of the original MS21 strain. Transformants were grown for 3 vegetative cycles on selective and non selective media (YNB or YNB/leucine, respectively). It was observed that in the medium supplemented with leucine, where there was no selective pressure, the transforming phenotype was lost rapidly, whereas in the selective medium the transforming phenotype remained. This mitotic instability of the *leu+* phenotype indicates that pCS5.1(3) is maintained extrachromosomally in the *Mucor* transformants. One pCS5.1(3)-transformant of each strain (MS7, MS8 and MS21) was selected for further analyses, and will be referred to as T7, T8 and T21, respectively.

**Figure 4:**
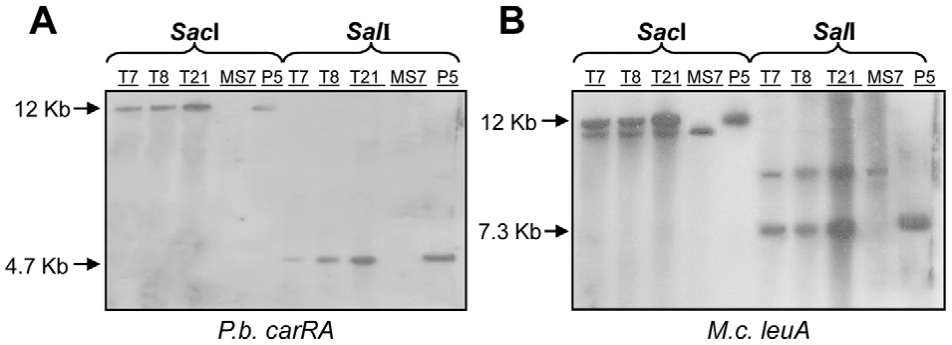
Southern blot analysis of the *Mucor* transformants. Genomic DNA from *M. circinelloides* untransformed MS7 strain was employed as negative control and plasmid DNA from pCS5.1(3) (P5 lane) was employed as positive control. DNA samples were digested with *Sac*I or *Sal*I enzymes. Hybridizations were performed with a *P. blakesleeanus carRA* probe (A) and a *M. circinelloides leuA* probe (B).

The presence of pCS5.1(3) in T7, T8 and T21 was analyzed by Southern blot hybridization. Genomic DNA was extracted, digested with *Sac*I or *Sal*I and prepared for hybridization with probes for the *carRA* and *leuA* genes (see Materials and Methods section). pCS5.1(3) plasmid DNA and genomic DNA from untransformed strain MS7 were employed as positive and negative controls, respectively. Figure 4 shows the results of this analysis. In the hybridization with the *carRA* probe (Figure 4A), a single band was detected in all the transformants and in the positive control (pCS5.1(3) lane) corresponding to the 12-kb linearized plasmid in the *Sac*I digestions and to the 4.7-kb *Sal*I-*Sal*I fragment of pCS5.1(3) that includes the *carRA* gene in the *Sal*I digestions. In the negative control (lane MS7) no signal was detected. In the hybridization with the *leuA* probe (Figure 4B), both in the *Sac*I and in the *Sal*I panels, a single band of different size was detected in the negative control MS7 lanes and in the positive control pCS5.1(3) lanes corresponding, respectively, to the endogenous and the plasmid-derived copies of the *M. circinelloides leuA* gene. Both bands were detected in the T7, T8 and T21 lanes. These results confirm that plasmid pCS5.1(3), and so the *P. blakesleeanus carRA* gene, is present in these transformants. In addition, it is shown that the plasmid is maintained extrachromosomally and does not seem to have suffered any major modification in the transformants.

**Figure 5:**
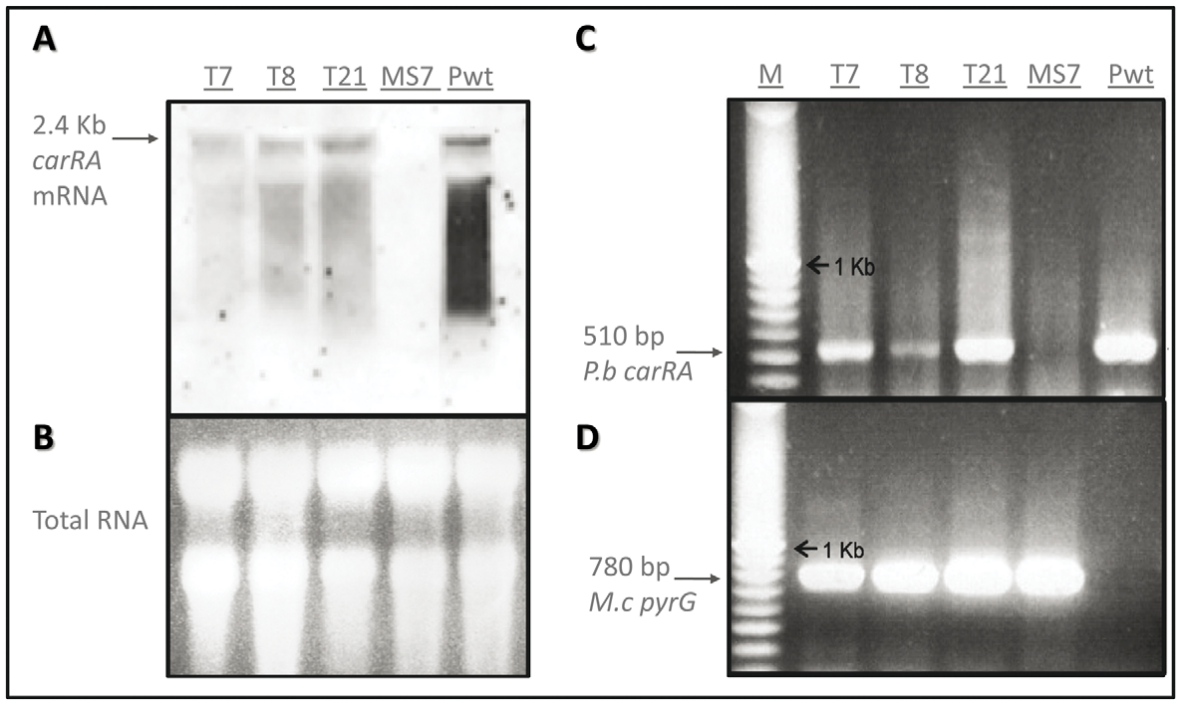
Northen blot and RT-RCR analyses of the *Mucor* transformants. A: Northern blot hybridization of total RNA from *M. circinelloides* transformants T7, T8 and T21 with a *carRA* cDNA probe. The untransformed *Mucor* MS7 strain and the *P. blakesleeanus* wild-type (Pwt) strain were used as negative and positive controls, respectively. B: Ethidium bromide staining after gel electrophoresis of RNA samples. C: Detection by RT-PCR of the *P. blakesleeanus carRA* cDNA in the same RNA samples. D: Quality control of the same RNA samples by RT-PCR detection of the *M. circinelloides pyrG* cDNA. M: 100 bp marker.

Transcription of the *P. blakesleeanus carRA* gene in the T7, T8 and T21 transformants was checked by Northern hybridization of total RNA using *P. blakesleeanus* wild-type strain and *M. circinelloides* untransformed strain MS7 as positive and negative controls, respectively (Figure 5A and B). While no hybridization signal was observed in the untransformed MS7 strain, a single hybridization band was detected in samples derived from transformants T7, T8 and T21. The size of this band corresponds to that of the band detected in the *P. blakesleeanus* wild-type lane. These results confirm that the *carRA* gene is expressed in these transformants. A clear mRNA smear is observed after hybridization with the *carRA* probe. This degradation tail has been previously observed in the analysis of the light-mediated expression of the *carB* and *carRA/P* genes of *Phycomyces* and *Mucor*, but not for other genes such as *actA*, *pyrF* and *pyrG* which expression is not light-regulated [45], [35], [48].

Furthermore, in order to confirm the correct expression and splicing of the *carRA* gene in the analyzed *M. circinelloides* transformants, a RT-PCR analysis was performed (Figure 5C and D). The same total RNA samples employed in the Northern blot assay were subjected to reverse transcription and the resulting total cDNA amplified with oligonucleotides PSP8 and PSP14, which had been designed to test the correct splicing of the *carRA* mRNA (see Materials and Methods section). A single amplification product of 510 bp was detected in T7, T8 and T21 transformants and the *P. blakesleeanus* wild-type samples, while no band was amplified from the untransformed MS7 strain (Figure 5C). These results further confirm that the *carRA* gene from *P. blakesleeanus* was expressed in these transformants and show that the intron in the *carRA* gene has been correctly processed in *M. circinelloides*. The quality of all the *Mucor* cDNA samples was checked by the positive control amplification of the *M. circinelloides pyrG* cDNA in the untransformed MS7 strain as well as in T7, T8 and T21 transformants, but not in the wild-type *P. blakesleeanus* strain (Figure 5D).

**Figure 6:**
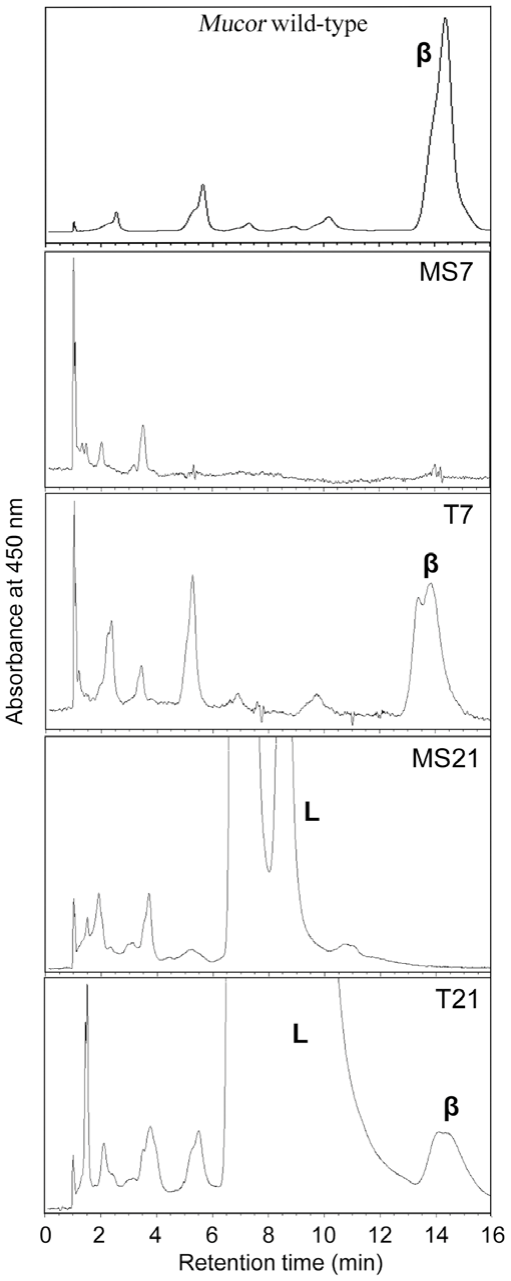
HPLC elution profiles of the carotenoids produced in the *Mucor* transformants. Carotenoids were detected at 450 nm. Profiles obtained from *M. circinelloides* wild-type, MS7 (*leuA1*, *carP4*) and MS21 (*leuA1*, *carR9*) strains, as well as T7 and T21 transformants (MS7 and MS21, respectively, transformed with the *P. blakesleeanus carRA* gene) are shown. β: β-carotene, L: Lycopene. Y-axis not scaled.

Finally, to study the functionality of the *Phycomyces carRA* gene product in the *M. circinelloides* T7, T8 and T21 transformants, an analysis of their carotenoid content was performed. Carotenoids were extracted from mycelia collected from the three transformants as well as from the untransformed strains MS7, MS8 and MS21 and analyzed by HPLC. Representative chromatograms of the obtained results are shown in Figure 6. As previously reported [33], [49], no β-carotene was detected in MS7, MS8 and MS21. However, small quantities of β-carotene were detected in the T7 (520±36 ng/gdw (g dry weight)), T8 (380±22 ng/gdw) and T21 (3252±19 ng/gdw) transformants (data are averages plus/minus standard errors of three independent experiments). Although these amounts are clearly lower than those observed in *M. circinelloides* wild-type (322 µg/gdw), the detection of β-carotene in the transformants indicates that the *carRA* gene can indeed complement, at least partially, the *M. circinelloides carR*, *carP* and *carRP* mutations.

### Complementation analysis in *E. coli*

Transformation of *E. coli* with plasmids carrying *crt* genes from *E. uredovora* leads to the synthesis and accumulation of different carotenoids in the bacteria [40]. In this system, a DNA fragment of 6.9 kb from the bacteria *E. uredovora* contains six open reading frames (*crt* genes) responsible for the transformation of GGPP into zeaxanthin-β-diglucoside. These genes are denoted as *crtE*, *crtX*, *crtY*, *crtI*, *crtB* and *crtZ*, and encode the enzymatic activities GGPP synthase, zeaxanthin glucosyl transferase, lycopene cyclase, phytoene dehydrogenase, phytoene synthase and β-carotene hydroxylase, respectively [40] (Figures 1 and 2). Elimination or inactivation of one or several of these genes allows the use of these *E. coli* “carotenoid mutants” for testing the function of a particular gene of interest by co-transformation of the corresponding cDNA and analysis of complementation of the pathway. As previously done for the homologous gene *carRP* from *M. circinelloides* [35], heterologous expression in *E. coli* was performed with the *Phycomyces carRA* cDNA to test if its encoded protein had phytoene synthase and lycopene cyclase activities. The results of these analyses are shown in Table 2.

**Table 2:** Carotenoid accumulation in *E. coli* co-transformants.

Plasmids in co-transformation a		Carotenoids b	
Cm	Amp	Lycopene	β-carotene
pAVB5 (*crtE*, *crtI*, *crtY*) [*crtB^−^*]	**+** pUC19	-	-
pAVB5 (*crtE*, *crtI*, *crtY*) [*crtB^−^*]	**+** pAVB13 (*crtB*)	-	116.71±20
pAVB5 (*crtE*, *crtI*, *crtY*) [*crtB^−^*]	**+** pCS19 (*carRA*)	11.18±2	4.22±2
pAVB12 (*crtE*, *crtB*, *crtI*) [*crtY^−^*]	**+** pUC19	181.54±32	-
pAVB12 (*crtE*, *crtB*, *crtI*) [*crtY^−^*]	**+** pAVB2 (*crtY*)	-	189.79±37
pAVB12 (*crtE*, *crtB*, *crtI*) [*crtY^−^*]	**+** pCS19 (*carRA*)	87.71±25	6.46±0.5
pAVB16 (*crtE*, *crtI*) [*crtB^−^*, *crtY^−^*]	**+** pUC19	-	-
pAVB16 (*crtE*, *crtI*) [*crtB^−^*, *crtY^−^*]	**+** pAVB2 (*crtY*)	-	-
pAVB16 (*crtE*, *crtI*) [*crtB^−^*, *crtY^−^*]	**+** pAVB13 (*crtB*)	129.89±9	-
pAVB16 (*crtE*, *crtI*) [*crtB^−^*, *crtY^−^*]	**+** pCS19 (*carRA*)	-	-
6pCS16 (*crtE*, *carB*) [*crtB^−^*, *crtY^−^*]^c^	**+** pUC19	-	-
6pCS16 (*crtE*, *carB*) [*crtB^−^*, *crtY^−^*]^c^	**+** pCS19 (*carRA*)	-	0.96±0.3

a Cm, chloramphenicol resistance; Amp, ampicillin resistance. Genes in plasmid are shown in brackets. “Carotenoid mutant” genotype is shown in square brackets.

b -, carotenoid not detected. All data are given in ng per gram dry weight. Data are averages and standard errors. Phytoene was not detected in any co-transformation.

c The gene for phytoene dehydrogenase from *E. uredovora* (*crtI*) has been replaced by the gene from *P. blakesleeanus* (*carB*), so this plasmid would be the same kind of “carotenoid mutant” as pAVB16.

**Figure 7:**
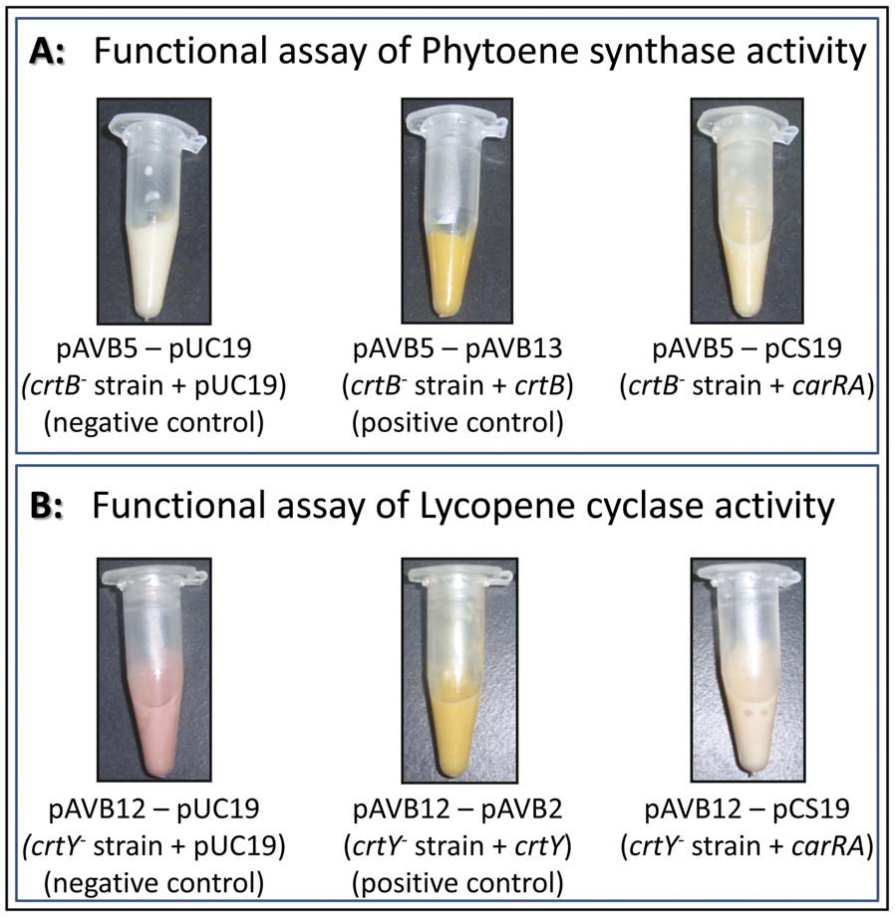
*E. coli* complementation assays. A: Phytoene synthase activity assay (*crtB^−^* strain; pAVB5). In the negative control (pUC19), bacteria do not accumulate any carotenoid and show a normal, whitish color. In the positive control (pAVB13), bacteria accumulate β-carotene and show a yellow phenotype. After transformation with pCS19, a light yellow color was detected (due to the β-carotene production as the result of the phytoene synthase activity from the *carRA* gene). B: Lycopene cyclase activity assay (*crtY^−^* strain; pAVB12). In the negative control (pUC19), bacteria accumulate lycopene and show a pink color. In the positive control (pAVB2), lycopene is converted into β-carotene resulting in a bright yellow color. Transformation with pCS19 led to a light-orange phenotype due to the β-carotene production as the result of the lycopene cyclase activity of the *carRA* gene. Data of HPLC analyses are shown in Table 2.

When the phytoene synthase (pAVB5 plasmid, *crtB^−^*) mutant was transformed with the *carRA* cDNA (pCS19), lycopene and β-carotene were produced, whereas no carotenoids were detected in the negative control (pUC19 co-transformation). Therefore, the *carRA* gene encodes an enzyme with phytoene synthase activity (Figure 7).

When the lycopene cyclase (pAVB12 plasmid, *crtY^−^*) mutant was transformed with the *carRA* cDNA, β-carotene was also produced though to a lower extent than with the positive control (pAVB2 plasmid carrying the *crtY* gene). However, in the negative control (pUC19 co-transformation) no β-carotene was detected. These results show that the CarRA protein also has lycopene cyclase activity (Figure 7).

Co-transformation assays using the phytoene synthase and lycopene cyclase double mutant (pAVB16 plasmid; *crtB^−^*, *crtY^−^*) were also performed. As expected, no carotenoids were detected with the negative control (pUC19) or the *crtY* (pAVB2) plasmids, while lycopene was accumulated after transformation with the *crtB* (pAVB13) plasmid. Co-transformation with the *carRA* plasmid pCS19, however, did not show any accumulation of carotenoids. It has been proposed that the enzymes encoded by the carotenogenic structural genes *carB* and *carRA* in *Phycomyces* form an enzymatic complex [4], [19]. We decided to check if the co-expression in *E. coli* of both *carB* and *carRA* cDNAs from *Phycomyces* had an impact on the efficiency of carotenoid accumulation. For this purpose, the *crtI* gene from plasmid pAVB16 was replaced by the cDNA from the *Phycomyces carB* gene to obtain the new plasmid 6pCS16, and similar co-transformation experiments were performed. The analysis of the carotenoids produced by these co-transformants showed that, although in very small quantities, β-carotene was obtained with plasmid pCS19 carrying the *carRA* cDNA. These results indicate that an inefficient interaction between the enzymes encoded by the *P. blakesleeanus* and the *E. uredovora* genes could be the cause of the low efficiency of the *Phycomyces* enzymes in this heterologous system.

## Discussion

The bifunctional lycopene cyclase/phytoene synthase gene is found only in fungi, and it is thought to have been originated from the fusion of two ancestral genes involved in lycopene cyclization (*crtYc* and *crtYd*) and a phytoene synthase gene [50]. The initial description of such a gene was carried out in *X. dendrorhous* (*crtYB*) [36]. The same situation was later reported for *M. circinelloides* (*carRP*) [35], *P. blakesleeanus* (*carRA*) [41] and *N. crassa* (*al-2*) [38]. As mentioned before, the *N. crassa al-2* gene had been previously described as encoding the enzyme phytoene synthase [37]. The insight gained with the fungal bifunctional gene description led to reveal *al-2* also as the lycopene cyclase gene by isolation of new mutants and sequencing of their *al-2* alleles [38] and by heterologous complementation in *E. coli* [39]. For *P. blakesleeanus*, it was already suggested that a bifunctional gene controls the lycopene cyclase activity, but the new reports [36], [35] led to a reconsideration of the second function associated with the *carRA* gene. The isolation of this gene and the sequencing of the allele in a number of mutants confirmed that it was altered in *carA*, *carR* and *carRA* strains [41]. The lycopene cyclase function had always been associated with the *carR* mutants. However, *carA* mutants had never before been associated with mutation in the phytoene synthase gene. The description of the CarRA product as an enzyme with lycopene cyclase and phytoene synthase activities was based on sequence homology with the proteins encoded by the genes mentioned above, but no actual functional analyses were available. We have addressed here this issue by complementation of *M. circinelloides* phytoene synthase and/or lycopene cyclase mutants as well as by heterologous complementation in *E. coli*.

The presence of β-carotene in the transformed strains of *M. circinelloides* and bacteria indicates that the *P. blakesleeanus carRA* gene does indeed encode a protein with both phytoene synthase and lycopene cyclase activities. Complementation of the *carP4* (strain MS7), *carRP5* (strain MS8), and *carR9* (strain MS21) mutations indicates that the *carRA* gene is not only correctly transcribed, but also correctly translated in *M. circinelloides*. Nevertheless, the amounts of β-carotene accumulated in T7, T8 and T21 transformants are very small when compared to those detected in the wild type strain or after transformation of the same strains with the homologous gene *carRP* [35]. An analogous heterologous complementation of the *Mucor* strain MS8 with the *carRA* gene of *Blakeslea trispora* has been described, also resulting in accumulation of small amounts of β-carotene in the transformants [51]. Similar low levels of carotenoid accumulation were reported when the *M. circinelloides* phytoene dehydrogenase mutant strain MS23 (*carB11*) was transformed with the *P. blakesleeanus carB* gene [23]. In that case the limited heterologous functional complementation achieved was attributed to the lack of specificity in the recognition of the initiation and termination transcription signals of the *Phycomyces carB* gene in *Mucor*, as mostly truncated *carB* transcripts were detected. A second explanation, which does not exclude the first one, is the fact that the formation of functional carotenogenic enzyme complexes in the transformants would be hampered by the simultaneous presence of both functional *Phycomyces* phytoene dehydrogenase subunits and mutant *Mucor* phytoene dehydrogenase subunits. In our case, the *Phycomyces carRA* gene seems to be correctly transcribed in *Mucor*. The particularly low β-carotene accumulation in T7, T8 and T21 transformants may be due to a low efficiency of the enzymatic activities of the protein/s expressed in this heterologous system. Therefore, alterations due to improper post-transcriptional or post-translational modifications, or failure to form a fully functional carotenogenic complex, are most likely responsible of the low level of complementation observed in these transformants. Integrative transformation is a rather uncommon event in *Mucor* [52]. The fact that the transformants obtained in this work do maintain the plasmid by autonomous replication and the coenocytic nature of the mycelium in this fungus, could also be a factor in the low levels of carotenoids detected. In our analysis of the *Mucor circinelloides carB* gene [45], we were able to compare the results of three different transformants, one of which resulted from integration of the transforming plasmid. This unusual case proved that β-carotene biosynthesis in that particular transformant was almost ten times higher than in the highest β-carotene-accumulating transformant with autonomous replication. It is probable that integration of the plasmid carrying the *Phycomyces carRA* gene would result in a higher level of β-carotene biosynthesis. However, even in this case, the accumulated amounts could still be very low and, therefore, it would be difficult to discriminate this kind of transformants from the majority of autonomous replicating transformants.

The expression of the *carRA* cDNA in the so-called *E. coli* “carotenoid mutants” led to accumulation of β-carotene when either *crtB* (phytoene synthase) or *crtY* (lycopene cyclase) genes were disrupted. The detected amounts were low but significant, and prove unequivocally the function of the CarRA protein. However, the same result could not be obtained when both *crtB* and *crtY* genes were altered; these kind of unfitting results are not uncommon, and we encountered something similar in our previous characterization of the *Mucor carRP* gene when complementation of the “*crtY* mutant” could be detected with a particular plasmid but not with a different one [35]. The low functional efficiency in complementation assays of *E. coli* “carotenoid mutants” is not surprising and has been reported in other cases. When bacteria deficient in the phytoene dehydrogenase activity were transformed with the *Phycomyces carB* and the *Neurospora al-1* cDNAs [42], very small amounts of carotenoids were detected, and it was attributed to translational or post-translational failures. We have shown here that co-expression of the cDNAs of both structural genes from *P. blakesleeanus*, *carB* and *carRA*, increases the efficiency of carotenoid biosynthesis in an appropriately constructed *E. coli* strain. Since the enzymes encoded by the carotenogenic structural genes in *Phycomyces* are supposed to work by forming an enzyme complex [4], [10], [19], it is not surprising that when this complex is formed in transformed *E. coli* “carotenoid mutants” from a pool of subunits encoded by genes from different organisms the efficiency of the system decreases.

All these observations demonstrate that the *P. blakesleeanus carRA* gene determines the phytoene synthase and the lycopene cyclase activities and suggest that both, species-specific post-transcriptional or post-translational modifications and particular specific protein-protein interactions between the *carB* and *carRA* gene products, are essential factors for the correct enzymatic activity of the carotenogenic complex synthesizing β-carotene in *P. blakesleeanus*.
